# Clinical evaluation of Dyslipidemia among type II diabetic patients at Public hospital Penang, Malaysia

**DOI:** 10.1186/1755-7682-3-34

**Published:** 2010-11-24

**Authors:** Nada F Zaki, Azhar S Sulaiman, Wasif S Gillani

**Affiliations:** 1 Dept of Clinical Pharmacy, University Sains Malaysia; 2 School of Pharmaceutical Sciences, University Sains Malaysia; 3 Dept of Clinical Pharmacy, University Sains Malaysia

## Abstract

**Background:**

Global views emphasize the need for early; effective intervention against the atherogenic dyslipidemia associated with type 2 diabetes and metabolic syndrome to reduce the risk of premature cardiovascular diseases. Our aim was to determine the clinical practices and compliance among dyslipidemia with type II diabetes and hypertension in multiracial society.

**Method(s):**

Study was carried out in out-patient department of General hospital Penang over a period of ten months (Jan - Oct 2008). Study reflects the retrospective data collection covering a period of three years from Jan 2005 - Dec 2007. Universal sampling technique was used to select all the patients' undergone treatment for diabetes type II and dyslipidemia. All the concerned approvals were obtained from Clinical research Committee (CRC). Data was analyzed by using SPSS 15^®^.

**Result(s):**

A total of 501 diabetes type 2 patients with dyslipidemia were identified in this study. The demographic data showed that 55.9% (n = 280) were female patients and 44.1% (n = 221) were males. Patients on combination therapy of metformin with other antidiabetic agent were 79%, while 21% were on monotherapy. Lovastatin was received as monotherapy in 83% of study population, while only 17% were on combination with gemfibrozil. Means of FPG and lipid profile were reduced from the initial (2005) to the latest level (2007) significantly (p < 0.001). Only 0.89% decrease in mean weight with S.D 13.1 as compared to initial S.D 12.8 after three years of Cohort. While in description 35.2% representatives gain weight with majority of males (71.5%), 52.3% with weight loss of 1-3 pounds majority (69.3%) with female respondents and rest 12.4% remains with same weight with mix gender distribution.

**Conclusion:**

Metformin and lovastatin use among patients of type 2 diabetes and dyslipidemia is significantly improved the clinical outcomes. No significant association of metformin or lovastatin is found against the hypertension. Metformin and calcium channel blocker combination therapy was found to be the best choice in the co-treatment of diabetes and hypertension.

## Introduction

Metformin is the only oral antidiabetic agent proven to reduce diabetes-related and total mortality in obese type 2 diabetes [1]. It has now been recommended as an agent of choice in the treatment of type 2 diabetes regardless of obesity [2]. Its predecessor, phenformin, was abandoned due to fatal cases of lactic acidosis. The use of metformin, with adherence to the contraindications and warnings, confirmed its safety in both post-marketing surveillance studies [3,4] and several clinical studies [5,6], where there was no excess risk of lactic acid associated with its use, compared to those not taking any agent. In usual practice, metformin has been widely prescribed for diabetic patients with a known contraindicated condition, but, apparently, no lactic acidosis cases were observed [7,8]. In fact, metformin use was associated with fewer deaths or hospitalizations in diabetes with heart failure [9,10]. On the other hand, cases of metformin-associated lactic acidosis continued to be reported sporadically [11-16]; concerns over the risk thus remain existence.

Although the causes of increased cardiovascular risks in both type 2 diabetes and metabolic syndrome are multifactorial, an atherogenic lipid profile characterized by elevated triglycerides and low levels of high-density lipoprotein (HDL) cholesterol are few major modifiable factor contributing progressively in cardiovascular risk [17]. Level of low-density lipoprotein (LDL) cholesterol typically is not elevated and in fact may even be below average. Although the size of the LDL particle is lower than average with the depletion of its cholesterol content, dense LDL does not contribute independently to the risk of CVD and is simply a marker for high levels of triglycerides and apolipoprotein B [18-20]. Thus be notable that the development of insulin resistance, dyslipidemia, and hypertension lead to the onset of overt glycemia and diabetes. During the latent phase before the clinical diagnosis of diabetes usually between 5-10 years, both macrovascular and microvascular complications were developed [21]. As a result, up to 50% of patients already have clinical evidence of macrovascular complications, and about 20% have microvascular complications at the time of diagnosis [22]. These findings emphasize the need for early; effective intervention against the atherogenic dyslipidemia associated with type 2 diabetes and metabolic syndrome to reduce the risk of premature CVD.

This study was aimed to characterize the clinical evidences in the use of hypoglycemic agents with Dyslipidemic drugs, in term to monitor laboratory parameters of Diabetes type II and Dyslipidemia. We assumed that both principal therapies (Metformin & Lovastatin) are significantly improving the core clinical symptoms. Study also intended to identify the best combination of hypoglycemic agents with antihypertensive medication to control hypertension with definitive diabetic control.

## Method(s)

A three years retrospective cross-sectional study was planned to achieve the above-mentioned objectives of the study. Study was carried out in out-patient department of General hospital Penang over a period of ten months (Jan - Oct 2008). During the data collection period, study deemed to cover three years cohort analyses which include 2005, 2006, 2007. Study population was targeted on the basis of type II diabetes with dyslipidemia and hypertension. Then the identified cohort of patients was analyzed against the respective monitoring parameters to identify the compliance and therapeutic effectiveness during a period of 3 years.

We took the continuous form of data for each monitoring parameter and compare and contrast against the mean and standard deviations against the normal range of standard hospital setting. All of the monitoring parameters were divided into two datasets', one includes the baseline reading on the initiation of the therapy and other was the last recorded value with the same therapy. If the therapy was changed from mono to combine therapy then the last lab values will be considered as the baseline value of combined-drug therapy and vice-versa. Patients' medication profile and laboratory tests for glucose and lipids were the prime monitoring parameters in this study. The study is certified with the approval of Clinical Research Committee (CRC) as all the ethical requirements were fulfilled during the study period.

Data obtained were analyzed by using the Statistical Package of Social Sciences (SPSS) version 15^®^, Chi-square, paired and independent t-tests as well as univariate analysis of variance (ANOVA) have been used to compare results. The level of significance was 0.05 with the confidence interval of 95%. All the results and findings were then illustrated in discussion section. We intend to find out the argument issues in this study to be benefited against the clinical practice.

## Result(s)

A total of all only 501 diabetes type 2 patients with dyslipidemia and hypertension were identified in this study by employing universal sampling. The demographic data showed that 55.9% (n = 280) were female patients and 44.1% (n = 221) were male patients. According to racial distribution, Chinese constituted 41.7% of the study population, Malay 34.3% and Indians 24%. The mean age was 62.2 ± 9.2 years. About 56.1% of patients were more and equal than 60 years old range, while 43.9% were in the less or equal range of 59 years. Lifestyle and social habits data demonstrated 86.2% non-smokers, 74.1% with uncontrolled diet and 78.4% on bad exercise. Patients on combination therapy of metformin with other antidiabetic agent were 79%, while 21% were on monotherapy.

Lovastatin was received as monotherapy in 83% of study population, while only 17% were on combination with gemfibrozil. Means of fasting plasma glucose (FPG) and lipid profile were reduced from the initial (2005) to the latest level (2007) significantly (p < 0.001) [23].

Progressive data analysis showed that 0.89% decrease in mean weight with S.D 13.1 as compared to initial S.D 12.8 over three years of time frame. While in description 35.2% representatives gain weight with majority of males (71.5%), 52.3% with weight loss of 1-3 pounds majority (69.3%) with female respondents and rest 12.4% remains with same weight with mix gender distribution. Fasting plasma glucose as a therapeutic monitoring of antidiabetic drug showed significant (p < 0.001) change of mean S.D value; towards low in the end of cohort as comparison initial base line values, but HbA1c value showed inconsistent results towards high mean S.D value in end of cohort with initial base line data. In contrast to metformin, lovastatin showed vice-versa better understanding against Low-density Lipoprotein and High-density Lipoproteins (table 1).

**Table 1:** Monitoring parameters in cohort of study

Parameters	Mean (SD)	N (%)
**Weight**		**501**
Initial weight	66.7 (12.8)	
Last weight	66.1 (13.1)	
Weight Change		
Increase	-	177 (35.3%)
Decrease	-	262 (52.3%)
No change	-	62 (12.4%)
**Fasting Plasma Glucose**		**501**
Initial	22.7 (116.3)	
During	8.4 (2.4)	
Last	8.2 (2.4)	
**HbA1c test**		
Initial	7.9 (1.8)	123 (24.6)
During	7.9 (1.6)	215 (42.9)
Last	8.2 (1.9)	194 (38.7)
**Total Cholesterol**		**501**
Initial	63.5 (232.1)	
During	63.4 (152.0)	
Last	25.1 (139.1)	
**Triglycerides**		**501**
Initial	65.8 (243.9)	
During	27.8 (158.6)	
Last	19.7 (132.6)	
**Low-density Lipoprotein**		**501**
Initial	101.1 (295.9)	
During	45.3 (199.7)	
Last	39.1 (185.5)	
**High-density Lipoprotein**		**501**
Initial	67.1 (247.7)	
During	31.2 (170.2)	
Last	21.2 (139.7)	
**Alkaline Phosphatase-ALP**		**501**
Initial	158.3 (262.1)	
During	124.4 (197.8)	
Last	104.3 (159.4)	
**Alanine Transferase - ALT**		**501**
Initial	111.3 (276.1)	
During	70.1 (204.4)	
Last	50.0 (155.7)	

Significant ineffective therapeutic outcomes were observed with uncontrolled FBS among combination of other antidiabetic drugs with metformin with p < 0.001 (table 2). Similarly better therapeutic compliance with monotherapy of Lovastatin was observed against the monitoring parameter of lowering LDL (89.9%) with increasing in HDL (94.7%) levels. While hypertension was significantly (p < 0.001) found among both monotherapy (77.9%) and combination therapy (76.5%) group (table 3). Finally our findings reflected that both metformin and lovastatin showed less medical complications with combination therapy or substitution with other therapeutic agents (table 4).

**Table 2:** Medication outcome with Metformin use.

Parameters (N = 501)	Metformin N(%)		
		
	Monotherapy	Combination	**χ**^**2 **^**value**
**Monitoring**			
Fasting Plasma glucose	**105 (20.9)**	**396 (79.1)**	0.001
Controlled	44 (41.9)	92 (23.2)	
Uncontrolled	61 (58.1)	304 (76.8)	
**Adverse Effects**			
Nausea	-	1 (0.3)	
Bloating	-	4 (1.0)	
Diarrhea	1 (0.9)	3 (0.8)	
Headache	-	1 (0.3)	
Weakness	-	4 (1.0)	
No adverse effects	**104 (99.1)**	**383 (96.6)**	0.023
**Hypertension complication**			
YES	86 (81.9)	303 (76.5)	0.001
NO	19 (18.1)	93 (23.5)	

**Table 3:** Medication outcome with Lovastatin use

	Lovastatin N(%)		
		
Parameters (N = 501)	Monotherapy	Combination	**χ**^**2 **^**value**
**Monitoing**			
**Total cholesterol**	**416 (83.0)**	**85 (17.0)**	0.001
High	40 (9.6)	12 (14.1)	
Normal	376 (90.4)	73 (85.9)	0.001
**Triglycerides**			
High	75 (18.0)	34 (40.0)	0.001
Normal	341 (82.0)	51 (60.0)	
**Low-density Lipoprotein**			
High	42 (10.1)	10 (11.8)	0.025
Normal	374 (89.9)	73 (88.2)	
**High-Density Lipoprotein**			
Low	22 (5.3)	5 (5.9)	0.031
Normal	394 (94.7)	80 (94.1)	
**ALT**			
High	34 (8.2)	10 (11.8)	0.16
Normal	382 (91.8)	75 (88.2)	
**ALP**			
High	46 (11.0)	5 (5.9)	0.54
Normal	370 (88.9)	80 (94.1)	
**Hypertension**			
YES	324 (77.9)	65 (76.5)	
NO	92 (22.1)	20 (23.5)	0.001
**Adverse Effects**			
YES	14 (3.4)	9 (10.6)	
NO	402 (96.6)	76 (89.4)	0.045

**Table 4:** Complications association with combination regimen among Cohort

Regimen design	Complications N/Total. (%)	χ^2 ^value ǂ
Metformin (monotherapy)	94/105. (89.5)	**0.001**
Lovastatin (monotherapy)	364/416. (87.5)	**0.001**
Sulfonlyurea	336/384. (87.5)	0.023
Insulin	70/73. (95.9)	**0.001**
Insulin & Oral hyperglycemic agent	61/64. (95.3)	0.001
Metformin & Diuretics	95/95. (100.0)	**0.000**
Metformin & Calcium Channel block	17/17. (100.0)	0.000
Metformin & Digoxin	2/2. (100.0)	0.000
Metformin & corticosteroid	9/9. (100.0)	0.000
Metformin & Nefidine	87/87. (100.0)	0.000
Lovastatin & Gemfibrozil	17/17. (100.0)	**0.000**
Lovastatin & other combinations	61/68. (89.7)	0.018

**ǂ **all the variables were in 2 × 2 type (as. Metformin = monoXcomb..etc), Similarly complications section is dictomise into (yes & no). Sig. value is taken on individual analysis latter compressed in this table

## Discussion

Patients with diabetes mellitus have a 2- to 4-fold increased risk of cardiovascular, peripheral vascular and cerebrovascular disease, which are the leading causes of morbidity and mortality in this population. Few epidemiological studies have shown an association between diabetic dyslipidemia, which is characterized by hypertriglyceridemia; low levels of high-density lipoprotein cholesterol; postprandial lipemia; and small, dense low-density lipoprotein cholesterol (LDL-C) particles - and the occurrence of cardiovascular disease. Metformin with thiazolidinedione/statins can be useful for the treatment of diabetic dyslipidemia [24].

Metformin works by increasing the number of muscle and adipocyte (fat cell) insulin receptors and the attraction for the receptor. It does not increase insulin secretion, it only increases insulin sensitivity. Therefore, metformin is not associated with causing hypoglycemia. This activity reduces insulin levels by increasing the sensitivity of peripheral tissues to the effects of insulin by rejuvenating the response, and restoring glucose and insulin to younger physiological levels that may cause weight loss and most certainly a decrease in the body's total fat content [24-29]. The moderate pattern of decrease in HbA _1c _during the mid phase of study has been shown to decrease total cholesterol and triglycerides, with significant change has been observed in HDL and LDL cholesterol [30].

It is often that patients with type II diabetes present with a cluster of cardiovascular risk factors like visceral obesity, hypertension, high triglyceride and low high density lipoprotein (HDL) cholesterol levels, and hypofibrinolysis, all of which form insulin resistance and potentially contribute to increased cardiovascular risk [30]. In the United Kingdom Prospective Diabetes Study, metformin was the only medication that reduced diabetes related deaths, heart attacks and strokes [15-20,31,32]. On the contrary, a few reports have documented a lack of effect of metformin on blood pressure [7-9,33]. Our study findings reflects the same identity of increase hypertension among both mono or combine therapy with metformin. So it is reviewed that long-term infusion with apparently nontoxic doses of metformin attenuates hypertension and decreases the hypotensive responses to ganglionic blockade in salt-induced hypertensive response (SHR), suggesting a centrally elicited sympathoinhibitory action [34].

A decrease in plasminogen activator inhibitor was most associated with the body weigh loss in subjects [31,35], but in our study no significant decrease in weight is observed whilst a significant pattern of weight gain is found. Probably these results suggested the non-exercise behavior with poor diet. In the observation of daily clinical practice, antidiabetic drug initiation for patients is sometimes delayed due to the potential risk of hypoglycemia, the need to educate patients on the use of antidiabetic drugs, the need to increase glucose control and concern over weight gain [11].

The multi-preventive effects of metformin on type II diabetes and evolving cardiovascular complications include a decrease in total cholesterol and low density cholesterol (LDL), free fatty acids, tissue plasminogen activator antigen and insulin levels when patients present with symptoms of hypertension, dyslipidemia, visceral obesity or hyperglycemia [31] in contrast to such reviews we find unusual high percentage of hypertension even with or without glycemic control and decrease in LDL and total cholesterol. Probably reason lies in two bare facts; first possibility suggest that due to increase of triglycerides (>2.25 mmol/L) with low LDL and HDL ratio [23-30] and second possibility is the weight gain with no-exercise behavior and poor diet control [11-13,17,24,31-34].

Long term use of metformin may cause malabsorption of vitamin B12 [21,25-27,31]. Because of the depletion of B12, supplementation is recommended [25-27]. When a person begins to take metformin, they may experience some nausea and vomiting, stomach pain, bloating and diarrhea [15,27]. Studies were conducted in the past that describe the pattern of oral antidiabetic and insulin use. These provide data on the level of glucose control achieved with each of these treatments, mono or combined, at different stages of the disease, the development of complications associated with DM2, the duration of therapy for each defined treatment pattern and clinical variables at the time the medication was changed [35-38].

## Conclusion

Metformin and lovastatin use among patients of type 2 diabetes and dyslipidemia is significantly improved the clinical outcomes. No significant association of metformin or lovastatin is found against the hypertension. Metformin and calcium channel blocker combination therapy was found to be the best choice in the co-treatment of diabetes and hypertension.

Hypertension is significantly found in both controlled and normal total cholesterol reading patients. This may reflects the attitude that lowering the FBS and total cholesterol can control hypertension, but for instance the hypertension among such patients have some inevitable cause to control.

## Competing interests

The authors declare that they have no competing interests.

## Authors' contributions

NFZ - Principal investigator; ASS - Manuscript editing and content evaluation; WSG - Manuscript writing and statistical analysis; All authors have read and approved the final manuscript.
